# Correlation between Endosonographic and Doppler Ultrasound Features of Portal Hypertension in Patients with Cirrhosis

**DOI:** 10.1155/2012/395345

**Published:** 2011-10-31

**Authors:** A. Wiechowska-Kozłowska, K. Zasada, M. Milkiewicz, P. Milkiewicz

**Affiliations:** ^1^Department of Endoscopy, Ministry of Internal Affairs Hospital, 70-382 Szczecin, Poland; ^2^Department of Radiology, M. Curie Hospital, Szczecin, Poland; ^3^Department of Laboratory Diagnostics and Molecular Medicine, Pomeranian Medical University, Szczecin, Poland; ^4^Department of Hepatology and Liver Transplantation, M. Curie Hospital, Szczecin, Poland; ^5^Liver Unit, Pomeranian Medical University, SPSK2, Szczecin, Poland

## Abstract

*Purpose*. Endoscopic ultrasound (EUS) permits the detailed visualization of clinically significant features of portal hypertension; however, it is an invasive procedure that is not widely available. The aim of this cross-sectional study was to determine whether a correlation exists between the features of portal hypertension detected using both Doppler ultrasound and EUS in subjects with liver cirrhosis. *Materials and Methods*. Analyzed cohort included 42 patients who underwent a detailed Doppler ultrasound focusing on the parameters of blood flow in the portal/splenic vein as well as an endoscopic/EUS procedure that included the assessment of the size and localization of “deep” varices. *Results*. The size of “deep” oesophageal varices detected with EUS exhibited no correlation with the parameters assessed by Doppler ultrasound. However, the size of the “deep” gastric varices detected using EUS correlated with the time averaged maximum velocity (T_max_ as well as V_min_, V_max_) for the portal vein using Doppler ultrasound and exhibited a correlation with the V_max_ and T_max_ for the splenic vein. No significant correlation was determined between the diameter of the azygous vein and the thickness of the gastric wall when seen on EUS versus the parameters measured with Doppler ultrasound. *Conclusion*. EUS provides important information regarding the features of portal hypertension, and in the case of “deep” oesophageal varices exhibits a limited correlation with the parameters detected by Doppler ultrasound. Thus, despite its invasiveness, EUS is a method that provides a reliable and unique assessment of the features of portal hypertension in patients with liver cirrhosis.

## 1. Background

Endosonography (EUS), a combination of both endoscopy and ultrasound, is a helpful tool for the assessment of portal hypertension in patients with cirrhosis [1–3]. However, gastroduodenoscopy remains the method of choice in the diagnosis of varices even though it only allows for the detection of varices of extrinsic (superficial) circulation of the oesophagus and stomach. EUS has a significantly higher sensitivity regarding the diagnosis of portal hypertension in comparison to gastroduodenoscopy and permits visualization of collaterals belonging to intrinsic (deep) venous circulation, which, if large, can significantly increase the risk of variceal bleeding [4–6]. Unfortunately EUS is an invasive procedure and remains not widely available [7–11]. On the other hand, Doppler ultrasound is a noninvasive method that provides precise information regarding blood flow in major vessels of the abdomen [12]. Doppler ultrasound is frequently used for the assessment of this aspect of portal hypertension. However, the potential relationship between the EUS and Doppler ultrasound results concerning portal hypertension have yet to be studied. The aim of this cross-sectional study was to establish whether the features of increased portal pressure detected with EUS show any correlation with the findings detected using Doppler ultrasound. 

## 2. Materials and Methods

Forty-two patients with cirrhosis referred to a tertiary liver centre were included in this study. The diagnosis was established on the grounds of liver biopsy and/or typical clinical features and imaging studies. At the time of EUS the following demographic and laboratory data were collected: age, gender, etiology of liver disease, liver biochemistry, platelet count, and Child-Turcotte-Pugh score (CTP score). 

All patients signed an informed consent form for the procedure. All examinations were done by two experienced endoscopists (AWK and PM), who had performed more than a thousand EUS procedures each. Endoscopy and EUS were performed as a single procedure using the GF-UMQ-130 echoendoscope (Olympus, Tokyo, Japan) at 7.5 and 12 MHz. Endoscopic features of portal hypertension were assessed first, followed by a detailed endosonographic examination of the stomach and oesophagus. The following data as part of the endoscopic examination were recorded: the presence and grade of oesophageal varices and the presence and grade of gastric varices. Oesophageal varices were graded as 0 = absent, small (<5 mm), and large (>5 mm) according to the most recent American Association for the Study of Liver Diseases (AASLD) guidelines. Gastric varices were graded as 0 = absent, 1 = small (<5 mm), or 2 = large (>5 mm). 

The following data were collected during EUS examination: oesophageal and gastric varices and oesophageal and gastric collateral veins (“deep varices”). A grade scale of three proposed by us previously [3, 11] was used for the assessment of “deep” varices depending on their size: grade 0: absent, grade 1: small <5 mm, grade 2: large >5 mm. The diameter of the azygos vein was measured within 2 cm above the level of the Z line, and the thickness of the gastric wall was assessed in the gastric cardia region as already described [3]. 

Doppler ultrasound analysis was performed using the Acuson XP unit (Acuson Mountain view, Calif., US) with a curved array 3.5–5 MHz transducer, and gray scale and color Doppler images were obtained. During the color Doppler examinations a low-volume flow filter with a high degree of motion discrimination was applied. Diameter, patency, and flow direction in the portal and splenic vein were assessed. This analysis included minimal (V_min⁡_  ) and maximal (V_max⁡_) flow in both analyzed vessels as well as T_max⁡_ (time averaged maximum velocity).

Statistical analysis was performed with ANOVA, chi-square, Yates, Fisher, and correlation coefficients using the StatView Program. *P* values < 0.05 were considered to be statistically significant.

## 3. Results

Basic demographic and clinical data on study subjects are summarized in Table 1. 

The size of the oesophageal varices exhibited a correlation with the diameter of the portal vein. In patients with grade 2 varices, this diameter was 13.6 ± 2.6 mm as compared to 11.1 ± 2.5 mm (*P* = 0.008) in subjects who had no varices, and 11.4 ± 2.4 mm (*P* = 0.04) in subjects with grade 1 varices. No statistically significant correlation was seen between the size of oesophageal varices and V_min⁡_, V_max⁡_, Tamx of both portal and splenic veins. Regarding the gastric varices seen on endoscopy, upon comparison of patients with grade 2 and grade 0 varices, the size showed a correlation with V_min⁡_ (17.0 ± 8.7 mm versus 11.8 ± 4.4 mm, *P* = 0.004). 

Data collected regarding the relationship between the grade of “deep” varices and the parameters of Doppler ultrasound are shown in Table 2. No statistically significant correlation between the size of “deep” oesophageal varices and the parameters recorded on Doppler ultrasound was seen. However, the size of “deep” gastric varices showed a correlation with V_min⁡_, V_max⁡_, and Tamx for the portal vein.

**Figure 1:**
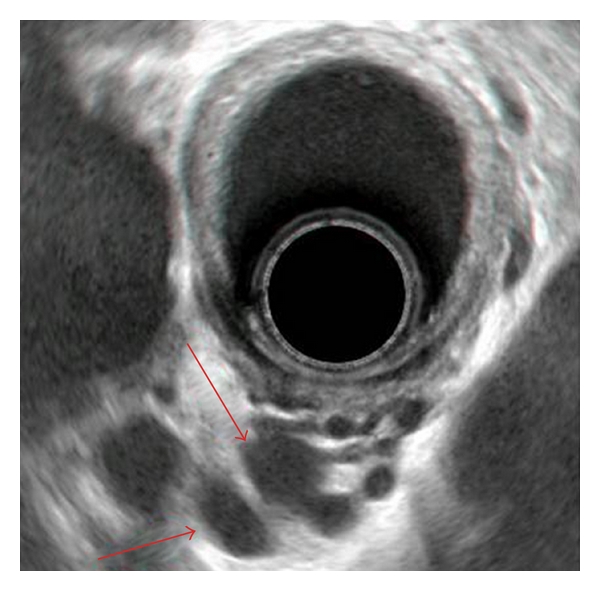
“Deep” oesophageal varices (red arrows) on endosonographic examination.

The correlation-coefficient analysis between the diameter of the azygous vein and the flow parameters in the portal and splenic veins showed no statistically significant correlation. Similarly, no significant correlation was seen between the thickness of the gastric wall and these parameters. These data are summarized in Table 3. A typical endosonographic image of large “deep” gastric varices is shown in Figure 1.

## 4. Discussion

Invasive angiographic technique such as HVPG (hepatic venous pressure gradient) measurement is frequently considered a gold standard in the study of the anatomy and pressures in patients with liver cirrhosis. Several groups have shown that patients with varices have a significantly higher HVPG than those without, but no clear correlation between HVPG and variceal size or bleeding risk has been firmly established [8]. Magnetic resonance (MR) imaging by means of time-of-flight or phase contrast angiography can both document the size and direction of the flow in studied vessels. Although this is a relatively easy method for the detection of spontaneous portosystemic collaterals, the pronounced variation within subjects raised reservation whether this technique will significantly contributes to the prediction of bleeding [8]. 

The advantage of EUS over upper gastrointestinal tract endoscopy in detection of features related to portal hypertension has been unequivocally shown in previous studies, demonstrating 92% sensitivity of EUS in the diagnosis of portal hypertension as compared to only 58% for upper GI endoscopy [1]. Despite this, EUS has not become a part of routine assessment for patients with liver cirrhosis, perhaps due to its limited availability and lack of properly designed prospective studies that utilize this modality for the assessment of patients with liver cirrhosis. EUS allows for visualisation of abnormal vessels belonging to intrinsic circulation, such as perioesophageal varices that are attached to the muscularis externa of the oesophagus and the paraoesophageal varices that are localized to the surrounding tissue [13]. Similarly, it allows for the detection of perigastric and paragastric varices [14]. They are often called “deep” varices and their presence is of prognostic value.

It has been previously demonstrated that patients with “deep” varices, a diameter exceeding 5 mm, are at higher risk of variceal recurrence after banding (93% versus 46%) and bleeding (43% versus 12%) [1, 15, 16]. In our previous study we have noted the presence of “deep” and potentially dangerous varices, which were undetected with routine endoscopy in a significant proportion of patients [3]. Thirty-three percent of subjects with large “deep” gastric varices showed no varices on endoscopy and 25% had only small ones [3]. Thus, identification of patients with large “deep” varices is of clinical importance. Also, an advanced hemodynamic study utilizing endoscopic color Doppler ultrasonography showed its potential usefulness in predicting recurrent variceal bleeding [17].

Abdominal Doppler ultrasound is a widely available, noninvasive tool that is a backbone in the assessment of patients with liver cirrhosis. The role of Doppler ultrasound in the assessment of clinically relevant features of portal hypertension remains controversial. Pleština et al. suggested that Doppler ultrasound may be of use in the prediction of the risk for oesophageal variceal bleeding [18]. However, these findings are inconsistent with the results of other studies. For example, Berzigotti et al. found that color Doppler ultrasound played no role in predicting clinically significant portal hypertension and oesophageal varices [19]. Also, Cioni et al. demonstrated the lack of a relationship between the parameters of portal flow and the risk of bleeding [20], while Li et al. demonstrated that Doppler ultrasound parameters of the portal vein exhibited no correlation with the advancement of endoscopic abnormalities in patients with cirrhosis [12]. 

We have a long-lasting interest in applying endoscopic ultrasound for the study of features of portal hypertension in patients with liver cirrhosis [2, 3, 11, 21]. In this study we aimed to determine whether simple measurements routinely assessed during Doppler ultrasound and which include flow parameters in the portal and splenic vein show any correlation with the features of portal hypertension detected with endoscopy and EUS. To our best knowledge, this is the first study that searched for a potential relationship between Doppler and EUS findings in these patients. 

We found that on endoscopy, the size of oesophageal varices correlated with the diameter but not with the flow parameters in the portal vein. 

We observed no correlation between the Doppler ultrasound findings and EUS regarding “deep” oesophageal varices. Thus, Doppler ultrasound does not seem to be an alternative, noninvasive tool in this respect. However, we also found that there was a significant correlation between the size of “deep” gastric varices and the flow parameters in the portal vein and a correlation with the flow in splenic vein. Thus, at least in the context of “deep” gastric varices, Doppler ultrasound findings could be of importance. Lack of a universal correlation between portal flow and the presence/size of “deep” varices should be interpreted in the context of an important role of hyperdynamic circulation in liver cirrhosis. Structural changes in cirrhotic liver leading to increased portal pressure are no longer considered a sole underlying cause of portal hypertension. Indeed, hyperdynamic circulation with increased cardiac output and decreased peripheral resistance leading to increased vascular flow may be responsible for the limited correlation between Doppler ultrasound and EUS findings. Additionally, hyperkinetic circulation may exert a negative effect on the decrease of portal pressure related to the development of collaterals.

In summary, this study demonstrated that EUS provides important information on portal hypertension in patients with liver cirrhosis that show limited correlation with basic flow parameters detected by Doppler ultrasound.

## Figures and Tables

**Table 1:** Demographic and clinical data of examined patients with liver cirrhosis (*n* = 42).

Age years (mean ± SD)	54 ± 12
Gender (M/F)	23/19
Etiology:	
(i) Viral and alcohol (*n*, %)	29 (69)
(ii) Autoimmune (*n*, %)	9 (21)
(iii) Cryptogenic (*n*, %)	4 (10)
Child A (*n*, %)	15 (36)
Child B (*n*, %)	23 (55)
Child C (*n*, %)	4 (9)

**Table 2:** Summary of Doppler ultrasound findings in relation to the size of “deep” oesophageal and gastric varices in the liver cirrhosis patients (*n* = 42). Data presented as mean ± SD. ^a^ *P* = 0.05; *P < 0.05; **P < 0.01 versus grade 0 varices.

Doppler US	“Deep” oesophageal varices size			“Deep” gastric varices size		
	0	1	2	0	1	2
V_min⁡_ portal	12.7 ± 5.4	13 ± 6.3	14.1 ± 7.1	10.0 ± 3.0	*16.1 ± 7.2	14.1 ± 6.8
V_max⁡_ portal	18.3 ± 5.3	20.6 ± 9.4	21.2 ± 10.2	14.8 ± 4.3	**25.0 ± 9.7	*21.3 ± 8.8
T_max⁡_ portal	14.8 ± 5.1	17.1 ± 9.0	17.7 ± 9.0	11.8 ± 3.2	**20.6 ± 7.9	*17.9 ± 9.0
V_min⁡_ splenic	14.6 ± 5.4	17.6 ± 9.6	16.9 ± 9.7	13.2 ± 5.7	17.7 ± 9.3	17.9 ± 9.5
V_max⁡_ splenic	21.8 ± 6.4	26.2 ± 14.4	25.1 ± 10.4	19.4 ± 5.8	^ a^ **27.6 ± 10.4**	^ a^ **26.1 ± 12.3**
T_max⁡_ splenic	17.3 ± 4.7	22.3 ± 11.0	20.1 ± 10.1	15.4 ± 5.0	^ a^ **22.2 ± 8.9**	^ a^ **21.6 ± 10.7**

**Table 3:** Correlation coefficient between the diameter of azygous vein/thickness of gastric wall and flow parameters examined in the patients with liver cirrhosis (*n* = 42).

feature	Coefficient for correlation	*P *value
Azygous vein diameter versus V_min⁡_ portal	0.085	0.62
Azygous vein diameter versus V_max⁡_ portal	0.170	0.32
Azygous vein diameter versus T_max⁡_ portal	0.162	0.34
Azygous vein diameter versus V_min⁡_ splenic	−0.026	0.88
Azygous vein diameter versus V_max⁡_ splenic	0.009	0.95
Azygous vein diameter versus T_max⁡_ splenic	0.022	0.89
Gastric wall thickness versus V_min⁡_ portal	0.030	0.85
Gastric wall thickness versus V_max⁡_ portal	−0.86	0.59
Gastric wall thickness versus T_max⁡_ portal	−0.49	0.76
Gastric wall thickness versus V_min⁡_ splenic	−0.202	0.20
Gastric wall thickness versus V_max⁡_ splenic	−0.163	0.31
Gastric wall thickness versus T_max⁡_ splenic	−0.129	0.43
